# Comparing two relaxation procedures to ease fatigue in multiple sclerosis: a single-blind randomized controlled trial

**DOI:** 10.1007/s10072-023-07042-x

**Published:** 2023-09-12

**Authors:** Guadalupe Garis, Christian Dettmers, Andrea Hildebrandt, Thomas Duning, Helmut Hildebrandt

**Affiliations:** 1 Present Address: Department of Psychology, Carl Von Ossietzky University Oldenburg, Oldenburg, Germany; 2 Department of Neurology, Klinikum Bremen-Ost, 28325 Bremen, Germany; 3 Kliniken Schmieder, Constance, Baden-Würtemberg, Germany

**Keywords:** Multiple sclerosis, Fatigue, Progressive muscle relaxation, Biofeedback, Autonomic nervous system, Heart rate variability

## Abstract

**Background:**

Various relaxation procedures have been proposed to reduce fatigue in multiple sclerosis (MS). However, it is unknown, which type of relaxation has the largest effect on fatigue reduction and on autonomic nervous system (ANS) activity.

**Objective:**

We aimed to compare two biofeedback-supported relaxation exercises: a deep breathing (DB) exercise and progressive muscle relaxation (PMR), which may ameliorate MS fatigue and alter ANS activity.

**Methods:**

We performed a single-blind randomized clinical trial, introducing MS patients (*n* = 34) to the DB or PMR exercise. We first tested cardiovagal integrity, reflected by changes in heart rate variability (HRV) in response to DB. Participants then performed a fatigue-inducing vigilance task, followed by the DB or PMR. State fatigue was recorded consecutively at baseline, after the vigilance task, and after the relaxation exercise, along with HRV reflecting ANS activity.

**Results:**

Only patients assigned to the PMR group experienced a significant drop in fatigue, whereas both relaxation exercises changed ANS activity. MS patients showed the expected autonomic response during the cardiovagal reflex test. The vigilance task elevated short-term feelings of fatigue and significantly reduced HRV parameters of parasympathetic activity. Trait fatigue was negatively correlated with HRV during the second half of the vigilance task.

**Conclusion:**

PMR alleviates short-term feelings of fatigue in persons with MS. The vigilance task in combination with HRV measurements may be helpful for evaluating relaxation procedures as a treatment of fatigue. Hereby, future studies should ensure longer and more frequent relaxation exercises and focus on patients with weak to moderate fatigue.

**Trial registration:**

Trial Registry: DRKS00024358.

## Introduction

Fatigue is one of the most frequent symptoms of multiple sclerosis (MS) [1] and is often defined as a subjective feeling of lack of physical and/or mental energy [2] that seriously interferes with a patient’s daily activities. Furthermore, it is often followed by a feeling of discomfort and decreased motivation [3]. Literature on fatigue has made the distinction between two forms of fatigue: trait fatigue, indicated by the propensity to fatigue over an extended period of time, and state fatigue, which refers to the acute experience of fatigue [4]. Having sufficient energy to perform daily activities is paramount to an individual’s well-being, and for that matter, fatigue can be the most distressing aspect of the disease [5–7].

Overall, knowledge toward an effective treatment of MS fatigue remains incomplete [8]. Commonly prescribed medications for decreasing MS fatigue like amantadine, modafinil, and methylphenidate are no longer advocated given that they were shown not to be more effective than placebo in improving MS fatigue [8]. Additionally, they cause adverse events more frequently [9], and are associated with long-term safety concerns [9].

Psychological interventions such as structured fatigue management programs, cognitive behavioral therapy, energy conservation interventions, and a mindfulness-based intervention may be beneficial [10, 11]. One important aspect of structured fatigue management programs (as a psychoeducational intervention) is learning and implementing relaxation techniques. A common type of relaxation technique, progressive muscle relaxation (PMR), is an exercise that includes voluntary stretching and relaxation of large muscle groups in the human body from hands to feet [12]. PMR has been found effective in reducing fatigue in MS patients at week 12, and its beneficial impact was still observed at follow-up, 2 weeks after the end of the intervention [13]. Other studies have similarly revealed a positive effect of PMR in decreasing MS fatigue [14, 15]. In contrast, a study by Sander et al. (2020) [16] found that biofeedback-supported PMR did not end with an instant reduction of state fatigue in MS patients.

Exercise therapy seems to ease symptoms of fatigue in MS [17]. Similarly, a recent review illustrated that yoga practices ameliorate symptoms of fatigue in MS patients when compared with usual care [18] indicating a temporary increase in parasympathetic nervous system (PANS) activity. It remains however unclear which element of yoga practices led to decreases in fatigue, since most reviewed studies involved a synergistic combination of different components of the yoga practice (movement sequences, stretching, breathing, meditation exercises). Thus, there is no distinct emphasis and specific research on one component or the other, limiting the interpretability of the results. Nevertheless, studies that integrated breathing techniques in the practice of yoga were significantly associated with decreased symptoms of fatigue [19–23], pointing to the beneficial effect of breathing exercises on MS fatigue.

Biofeedback may be used to increase the effectivity of relaxation training, as it informs about increases in PANS activity [24, 25]. In biofeedback learning, information on bodily functions is visualized on a screen to help the person learn how to modulate these functions [26]. Increases in heart rate variability (HRV) parameters of PANS activity have been observed in relation to biofeedback-assisted relaxation in patients with hypertension [27], as well as healthy participants [28]. Through enhanced parasympathetic control, biofeedback would also be expected to result in reduction of fatigue. However, a causal relationship cannot be yet assumed, as biofeedback could nevertheless activate other systems (cognitive or limbic) that may also be influencing PANS. Given that autonomic reactivity can be impaired in MS patients [29–31], it remains unclear if biofeedback can be used in these patients.

In the current study, we aim to use biofeedback to train two different relaxation strategies and to analyze their effect on MS-related fatigue. For that purpose, we first analyze whether an effect of deep breathing (DB) on PANS activity (the cardiovagal reflex) can be measured by changes in HRV, which has to be shown before using biofeedback as part of a treatment strategy. We then carried out a single-blind randomized controlled trial with two groups of MS patients. The first group participated in a deep breathing (DB) exercise, whereas the second group practiced a variant of PMR treatment that has been linked with increased parasympathetic activity (based on the results of Sander et al., 2020) [17]. Both groups first learned to practice either of the relaxation strategies, then performed a vigilance task that was aimed to increase their current (state) feelings of fatigue. Shortly afterward the patients were requested to carry out the relaxation strategies that were aimed at alleviating their fatigue.

We measured subjective feelings of fatigue in response to the vigilance task and the relaxation exercises. We further explored the association between the feeling of fatigue and the biofeedback measures. For that purpose, we measured HRV throughout the fatigue-inducing task and after practicing the relaxation techniques.

Given the study design, we expected that the DB group would bring about higher recovery from fatigue than the PMR group after the fatigue-inducing vigilance task. We further expected that the vigilance task would be related with decreased PANS activity, whereas the relaxation exercises would increase PANS activity.

## Methods

### Participants

Forty MS patients fulfilling inclusion criteria were recruited at the Kliniken Schmieder in Constance (Baden-Württemberg, Germany) or were otherwise referred from self-help groups in Oldenburg (Niedersachsen, Germany) and surroundings. Participants were required to have a clinical diagnosis of MS according to the McDonald criteria [32], be older than 18 years, have a clinical diagnosis of MS, no relapse or corticosteroid use in the previous 4 weeks, no psychoactive medication use (such as noradrenergic antidepressants, modafinil, and amantadine), and no MS-independent psychiatric disease. We have also asked for the presence of ischemic heart disease, lung disease affecting the ability to perform the deep breathing exercise to prevent adverse side effects. Available information on immunomodulatory drugs and disease progress was collected from the medical record (e.g., Expanded Disability Status Scale (EDSS) [33], disease duration). If this was not available, the Patient-Reported Expanded Disability Status Scale was administered instead. Our participants reported whether they were using the following disease-modifying treatments: Copaxone, teriflunomide, Tecfidera, interferon beta-1a, ocrelizumab, natalizumab, and fingolimod. The study was approved by the medical ethics committee of the University Medicine Oldenburg (UMO) (approval number 2020–152) and was registered in the German Clinical Trials Register (registration number DRKS00024358). Written informed consent was obtained from each participant before initiation of the study. Group characteristics are summarized in Table 1.

**Table 1:** Characteristics of the patient groups

	Total sample	Deep breathing group	Progressive muscle relaxation group
Sex (female/male)	(23/11)	(10/7)	(13/4)
MS course (RRMS/PPMS/SPMS)	(21/8/5)	(9/4/4)	(12/4/1)
Recruiting place (Oldenburg/Konstanz)	(14/20)	(6/11)	(8 / 9)
	*M* ± *SD*	*M* ± *SD*	*M* ± *SD*
Age (years)	46.2 ± 12.5	47.4 ± 13.9	44.9 ± 11.3
Disease duration	12.3 ± 8.2	12.1 ± 9.3	12.4 ± 7.2
Expanded Disability Status Scale	3.4 ± 1.8	3.6 ± 1.4	3.1 ± 2.1
Multiple Sclerosis Functional Composite	− 2.0 ± 2.5	− 2.3 ± 1.9	− 1.8 ± 3.0
Beck Depression Inventory	10.5 ± 6.7	9.8 ± 5.3	11.2 ± 8.0
Pittsburgh Sleep Quality Index	6.2 ± 3.6	6.2 ± 3.9	6.2 ± 3.4
Epworth Sleepiness Scale	9.1 ± 3.8	9.3 ± 3.9	8.8 ± 3.8
Fatigue Scale for Motoric and Cognition	72.7 ± 13.7	75.5 ± 15.9	69.8 ± 10.9
State fatigue pre vigilance task	32.4 ± 18.8	33.1 ± 14.6	31.8 ± 22.7
State fatigue post vigilance task	56.9 ± 20.4**	56.2 ± 21.1*	57.6 ± 20.4*
State fatigue post relaxation	43.3 ± 24.9**	47.5 ± 24.9	39.0 ± 24.9*

*RRMS* relapse-remitting MS; *PPMS* primary progressive MS; *SPMS* secondary progressive MS. No statistically significant differences between the two intervention groups. * *p* < 0.05; ** *p* < 0.01 for intragroup comparisons

**Fig. 1:**
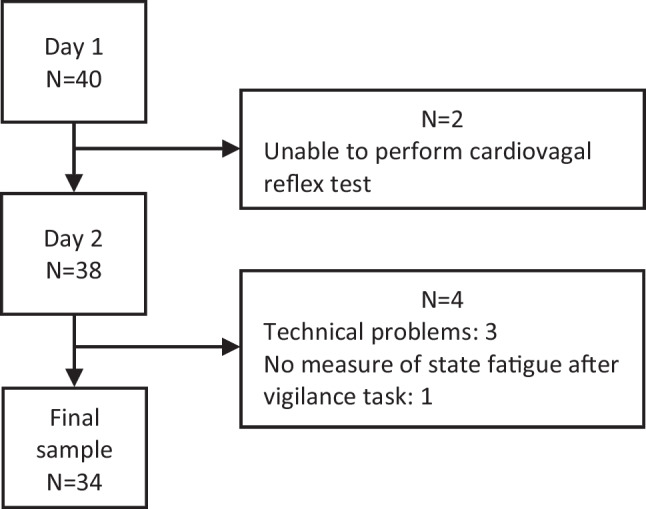
Flowchart illustrating the dropouts

After the dropout of 6 patients, the final study group consisted of 34 MS patients (see Fig. 1. for dropout reasons). The PMR group and the DB group consisted of 17 participants each.

### Design

The study was a single-blind, randomized controlled trial with two arms: (a) the DB group, performing the deep breathing exercise, and (b) the PMR group, being instructed to relax muscles on distal body parts, involving the muscles of the arms, shoulders, legs, and feet. This study was designed (a priori) as a superiority trial with a null effect expected on the PMR group, based on the findings from Sander et al. [17]. Subjects were assigned to either of the groups by a computer-generated simple block randomization method. An investigator that was not involved in data collection prepared the assignment of participants in A4 paper sheets that were twice folded in half. The main investigator knew which group a participant belonged to by opening a paper sheet with a label “true” (referring to the intervention group, that is, DB) or “false” (referring to the active control group, that is, PMR), indicating the allocation group, so as to prevent “selection bias.” In addition, the main investigator kept participants unaware of group allocations.

### Procedure

**Fig. 2:**
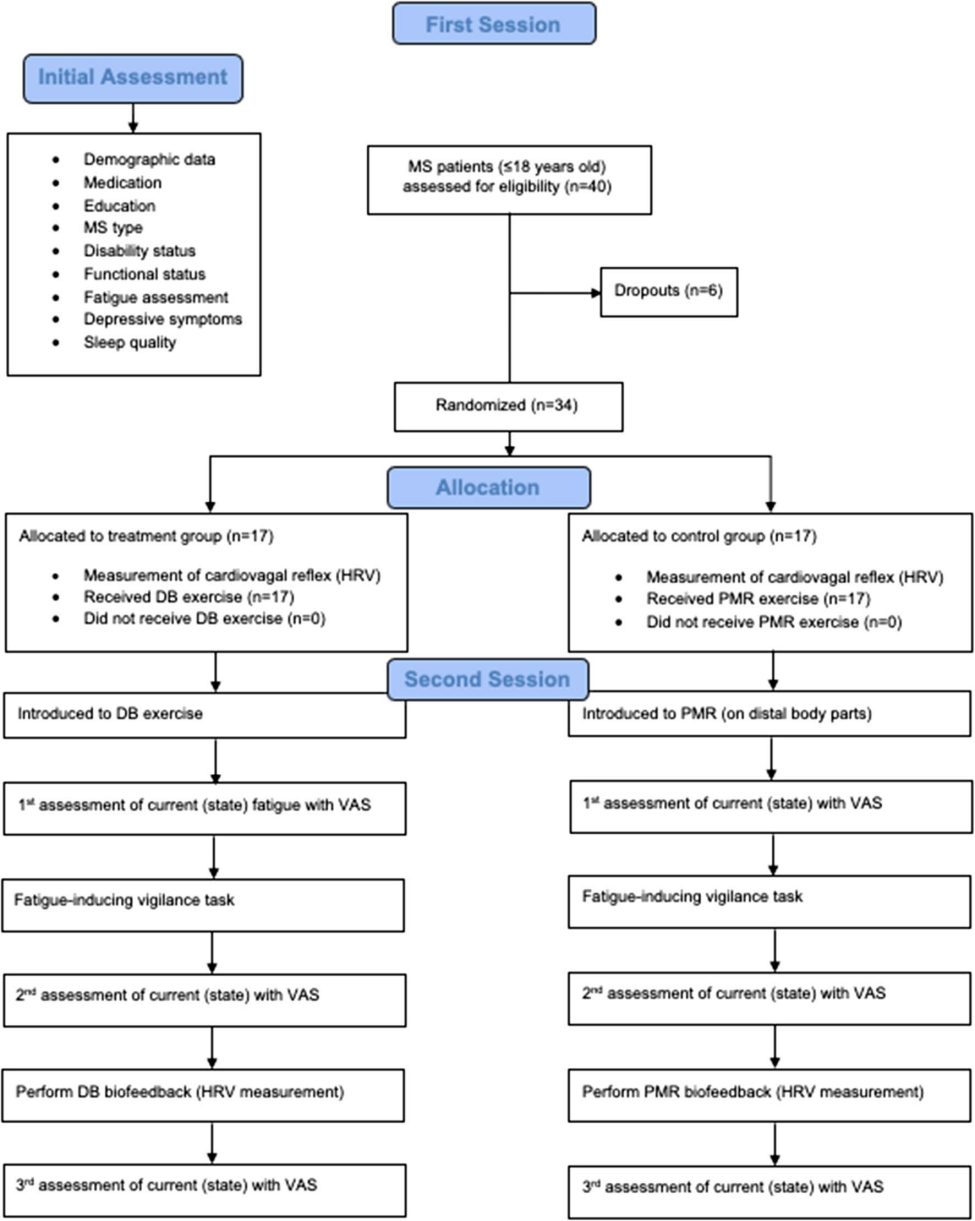
CONSORT diagram showing the flow of participants throughout the trial

The assessments and the relaxation exercises were performed in a quiet room at two different times on the same day. An evaluation of the ANS by means of HRV was carried out during the whole procedure. Before each task, the instructions were read out loud to the participant from a protocol sheet. See Fig. 2 for CONSORT diagram that represents the participant flow through the trial.

In the first session, which took place in the morning, participants filled out questionnaires, followed by two cardiovagal reflex tests (reflected by HRV levels in response to DB). Participants rested for a minute before the cardiovagal reflex test. The test involved participants sitting quietly and being instructed to breathe slowly and smoothly at a frequency of five cycles per minute (5 s of inhalation and 5 to 8 s of exhalation). The first assessment of HRV was obtained during baseline condition with normal breathing. Participants were then instructed to carry out the cardiovagal reflex test at three separate consecutive times. The first test was meant as a learning session. HRV was subsequently recorded during the second and third cardiovagal reflex tests. Afterward, the investigator checked the group allocation of the participant and trained participants to the DB or PMR exercise (on distal body parts), for the purpose of familiarizing them with the task.

MS patients in the DB group were instructed to breathe continuously through the nostrils and with the mouth closed, with respiratory movements that should resemble a wave. They were also advised not to take sudden inhalations/exhalations or hold the breath to prevent hyperventilation. During the biofeedback sessions, participants were instructed to visualize their breathing rate and breathing depth on a computer screen.

MS patients in the PMR group were instructed to increase muscle tension in distal body parts vigorously for about 20 s each, but without really straining them, and then release this tension for separate muscles of those body parts. They were directed to rest on their back and concentrate on their body. Participants needed to tense up and release each distal body part three times consecutively. Specifically, participants were directed to tightly clench the right fist, bend their right arm, stretch their right arm by extending it, lift their shoulders as if they could touch their ears, tighten their thighs by squeezing their two knees together as if they were holding a coin between them and to flex their feet, and pull their toes toward themselves and feel the tension in their calves.

The PMR exercise did not involve muscles of the face and neck, and abdomen, as participants had to wear a protective face mask and a respiration sensor over the abdomen. Inhalation and exhalation patterns were displayed on the computer screen. Lastly, participants of both groups were asked not to smoke and drink coffee or black tea for at least 2 h prior to the second session. The first session lasted approximately 90 min.

The second session, which took place in the afternoon, on the same day as the first session, started with the rating of current (state) fatigue. Afterward, participants performed a fatigue-inducing and monotonous acoustic vigilance task from the Test Battery for Attentional Performance (TAP) [34] lasting 30 min. Current (state) fatigue levels were assessed again. Afterward, participants performed the DB or PMR exercise for 5 min, followed by a 5-min break.

Both relaxation exercises began with a short resting period that gave patients time to relax. Before starting each procedure, the investigator made sure heart rate was not trending up by ensuring a private and quiet experimental setting. As participants performed the exercise, they could visualize their respiration pattern and heart rate on a computer screen. Then, the current level of fatigue was rated for the last time to assess whether the relaxation exercises would positively impact fatigue recovery after the vigilance task. The second session lasted approximately 60 min.

### Assessment

The primary outcome, current (state) fatigue level, before the vigilance task, after the vigilance task, and after the relaxation exercise, was assessed with a visual analog scale (VAS). The Fatigue Scale for Motor Skills and Cognition (FSMC) was used as a measure of cognitive and motor trait fatigue in people with MS [35]. To assess depressive symptoms, the Beck Depression-Inventory (BDI) was administered [36]. Additionally, we applied the Patient-Reported Expanded Disability Status Scale (PREDSS) to quantify disability [37], and the Multiple Sclerosis Functional Composite (MSFC) [38] to assess clinical status. All three subtests of the MSFC were administered, that is, the Timed 25-Foot Walk (T25W) for assessing leg function, 9-Hole Peg Test (9HPT) for assessing arm and hand function, and the Paced Auditory Serial Addition Test (PASAT-3) for cognitive function [38]. In addition, the assessment of sleep quality and daytime sleepiness was carried out by the Pittsburgh Sleep Quality Index (PSQI) [39] and the Epworth Sleepiness Scale (ESS) [40], respectively.

We chose the monotonous low event rate vigilance task of the TAP as it is an experimental task that systematically triggers fatigability and was shown to be correlated with MS-related fatigue [41] due to the demanding nature of the sustained attention induced by the task. Beeps of 440 and 1000 Hz were played alternatingly with an inter-stimulus interval of 1.3 s. On 24 occasions, another stimulus of the same frequency was presented consecutively, and the participant had to press a button immediately after detecting such a beep. A 2-min practice session was administered prior to the task.

Heart rate was measured with the Blood Volume Pulse Sensor of the NeXus-4 Biofeedback-System of Hasomed. A sensor was placed on the index finger of the non-dominant hand of the patient. We chose to apply the Blood Volume Pulse Sensor rather than a standard electrocardiography (ECG) because it is a more convenient, quicker to install, and less intrusive method than a standard ECG. Breathing rate and relative depth of abdominal or thoracic breathing were measured with the Respiration Sensor of the NeXus-4 Biofeedback-System of Hasomed, provided with an adjustable elastic band worn over the abdomen and over clothing.

The beginning and end of the vigilance task, the cardiovagal reflex test, and its corresponding baseline preceded the test, and the relaxation exercises were marked with the NeXus Biotrace Software. Obtained interbeat (RR) intervals were further processed by using Kubios HRV Standard analysis software (version 3.5.0). Pre-processing was done with the threshold-based artefact correction algorithm. Artifacts were identified in the data, and the most suitable correction level (being the lowest possible to avoid overcorrection of the data) was selected. A medium threshold (0.25 s) artefact reduction was applied. The identified artifacts were replaced by interpolated values.

Based on previous results [42], we focused on specific measures in the HRV frequency-domain and time-domain which reflect PANS activity [43], that is, the root mean square of successive differences between normal heartbeats (RMSSD), and standard deviation of all interbeat intervals (SDNN).

### Statistical analyses

Statistical analyses were performed with SPSS 25 [44]. We checked the data for normal distribution with the Shapiro–Wilk test, and for Equality of Variances with Levene’s test. The Greenhouse–Geisser correction was used when the assumption of sphericity was violated for repeated measures analyses of variance. Differences between the intervention groups were compared using a *t*-test. Pearson correlation was used to analyze the relation between HRV data, demographic and baseline data, and trait and state fatigue levels. For post hoc testing, we used the Bonferroni correction for multiple comparisons.

#### Effect of the vigilance task and the relaxation group on state fatigue

We further tested the impact of the vigilance task and the relaxation intervention on current (state) fatigue, and whether any changes are modulated by the relaxation group. Hereby, Time (comprised by baseline state fatigue, state fatigue after the vigilance task and state fatigue after the relaxation intervention) served as the within-subjects variable, and Group (DB or PMR) served as the between-subjects variable.

#### Effect of deep breathing on heart rate variability

A one-way repeated measure analysis of variance (ANOVA) was conducted to analyze whether the cardiovagal reflex tests brought about expected changes in HRV parameters. Hereby, Time (comprised by baseline, first cardiovagal reflex test and second cardiovagal reflex test) served as the within-subjects variable on mean levels of HRV parameters (SDNN and RMSSD).

#### Effect of the vigilance task on heart rate variability

Another one-way repeated measures ANOVA served to test whether performing the vigilance task affected HRV levels. Hereby, Time (first half of the vigilance task, second half of the vigilance task) served as the within-subjects variable on mean levels of HRV parameters (SDNN and RMSSD).

#### Effect of the vigilance task on reaction time

To test whether practicing the relaxation techniques affected HRV levels, Time (second half of vigilance task and post relaxation exercise) served as the within-subjects variable, and Group (DB or PMR) served as the between-subjects variables on mean levels of HRV parameters (SDNN and RMSSD).

#### Effect of relaxation exercises on heart rate variability

Lastly, we tested whether reaction time changed throughout the vigilance task. Hereby, Time (first half of vigilance task and second half of vigilance task) served as the within-subjects variable, and Group (DB or PMR) served as the between-subjects variables on mean levels of reaction time (in milliseconds).

## Results

### Relaxation group characteristics

The DB and PMR groups did not differ in age, disease duration, disease course, functional (MSFC) and disability (EDSS) status, fatigue level (FSMC), depression (BDI), daytime sleepiness (ESS), and sleep quality (PSQI).

### Effect of the vigilance task and the relaxation intervention on state fatigue

**Fig. 3:**
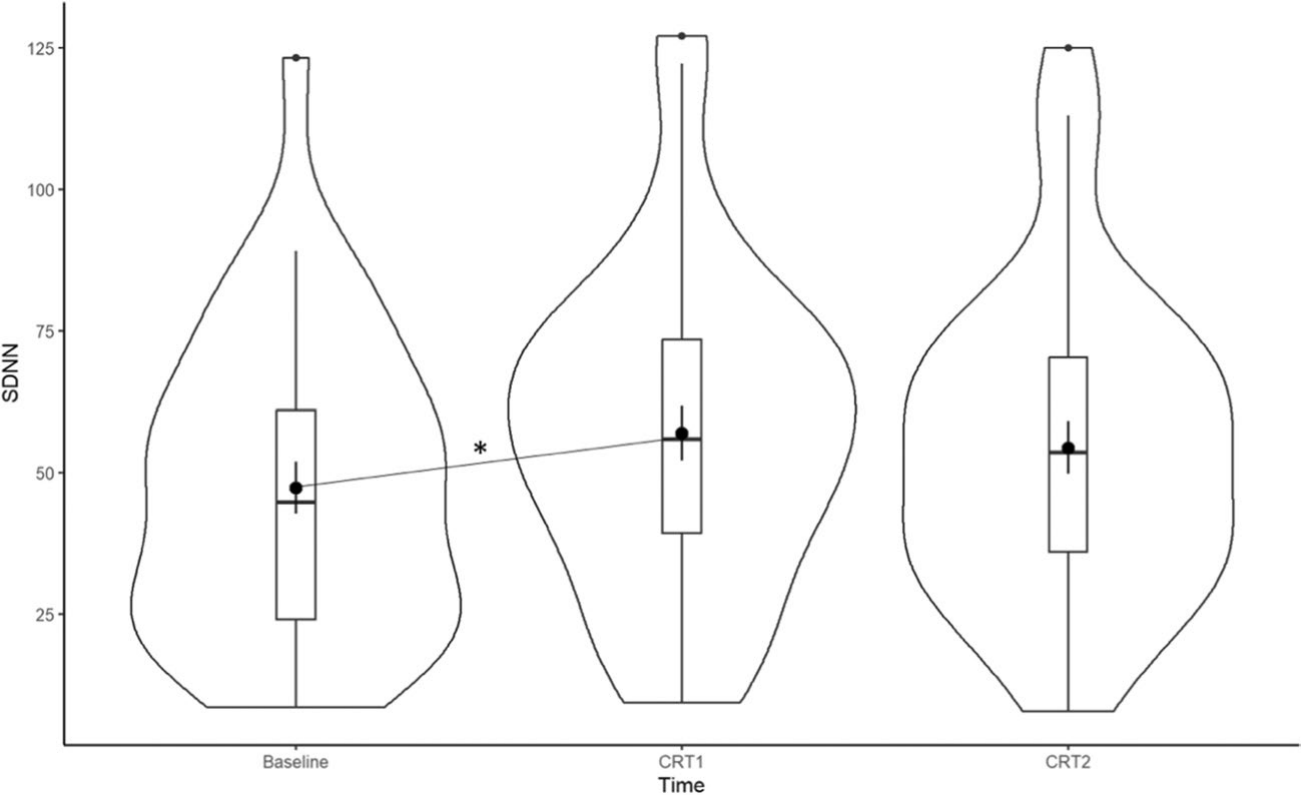
Violin plot showing density data of state fatigue at baseline, after the vigilance task, and after the relaxation exercise for the two relaxation groups. * *p* < 0.05; ** *p* < 0.01. *Note*. PMR.B, during baseline in the progressive muscle relaxation group; DB.B, during baseline in the deep breathing group; PMR.PT, after vigilance test in the progressive muscle relaxation group; DB.PT, after vigilance test in the deep breathing group; PMR.PR, after relaxation exercise in the progressive muscle relaxation group; DB.PR, after relaxation exercise in the deep breathing group

The relaxation groups did not differ on state fatigue during baseline, before the vigilance task began. A main effect of Time was found on state fatigue [*F*(2,64) = 23.172, *p* =  < 0.001]. Post hoc independent *t*-test revealed that experienced state fatigue increased significantly after performing the vigilance task when compared to baseline [Δ = 24,456, *p* =  < 0.001], and decreased after carrying out the relaxation exercise [Δ = -13.597, *p* = 0.004]. In this respect, the PMR exercise led to a statistically significant drop in state fatigue [Δ = -18.561, *p* = 0.005], while the DB exercise did not (Fig. 3, Table 2). However, there was no significant Group effect or Group * Time interaction.

**Table 2:** State fatigue, SDNN, RMSSD and reaction time for the treatment groups

Groups:	DB	PMR
	Mean ± SD	Mean ± SD
State fatigue: after vigilance task	56.21 ± 21.18	57.64 ± 20.41
State fatigue: after intervention	47.58 ± 24.94	39.08 ± 24.92*
SDNN: during vigilance task	44.63 ± 22.09	47.68 ± 31.20
SDNN: after intervention	53.42 ± 31.85*	61.82 ± 46.89*
RMSSD: during vigilance task	45.91 ± 26.54	49.72 ± 33.37
RMSSD: after intervention	56.63 ± 38.49	54.77 ± 32.54
Reaction time: first half vigilance task	781.87 ± 312.41	676.43 ± 135.65
Reaction time: second half vigilance task	851.21 ± 377.68	749.31 ± 147.93

*DB* deep breathing; *PMR* progressive muscle relaxation

*p < 0.05 for intragroup comparisons

### Effect of deep breathing on heart rate variability

**Fig. 4:**
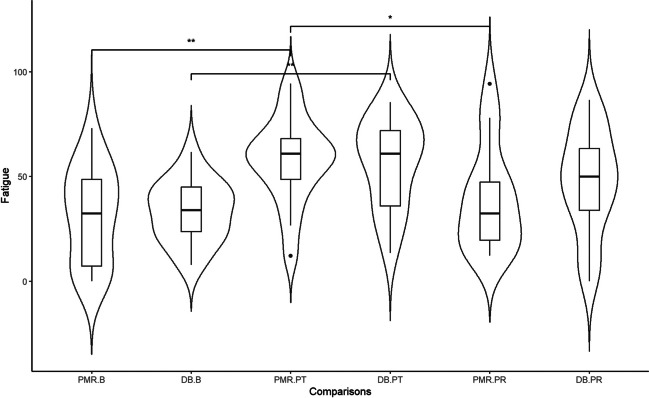
Violin plot showing density data of SDNN at baseline, during the first cardiovagal reflex test, and during the second cardiovagal reflex test. * *p* < 0.05. *Note*. CRT1, first cardiovagal reflex test; CRT2, second cardiovagal reflex test

A main effect of Time was found on SDNN [*F*(1.336, 64) = 4.004, *p* = 0.041]. SDNN was higher during the first cardiovagal reflex test, followed by the second cardiovagal reflex test and during the baseline (Fig. 4). RMSSD did not differ statistically significantly between Time points.

### Effect of the vigilance task on heart rate variability

No main effect of Time was found on SDNN and on RMSSD for the vigilance task.

### Effect of the vigilance task on reaction time

A main effect of Time was found on reaction time [*F*(1,30) = 15.273, *p* =  < 0.001]. Reaction time was higher during the second half of the vigilance task than during the first half of the vigilance task (Table 2).

### Effect of relaxation exercises on heart rate variability

There was a main effect of Time on SDNN [*F*(1,32) = 6.612, *p* = 0.015]. Post hoc analysis showed that patients had significantly higher SDNN levels following the relaxation exercise than prior to it [Δ = 11.465, *p* = 0.015] (Table 2). A main effect of Time was not found on RMSSD. Post hoc analysis showed that patients also had significantly higher RMSSD levels because of the relaxation exercise than prior to it [Δ = 14.101, *p* = 0.003]. There was no significant Group effect or Group * Time interaction, demonstrating no significant difference between the DB and PMR groups in their HRV levels following the relaxation exercise.

### Sub-group analysis

Autonomic disorders and HRV may differ according to disease course in MS [45]; therefore, MS disease course–specific subgrouping is of interest. Table 1 shows that 21 MS patients with relapse-remitting disease course were included in the study, 8 with primary progressive, and 5 with secondary progressive. The small number of progressive patients in each group did not allow for sub-group analyses. In RRMS patients, deep breathing elicited an increase in SDNN levels [*F*(1.436) = 5.420, *p* = 0.017]. Post hoc testing showed that both interventions differed significantly from baseline SDNN. However, there was no effect of deep breathing on RMSSD.

Vigilance testing induced higher levels of fatigue, which dropped significantly after PMR (using intragroup comparison), and not after DB. The relaxation interventions increased SDNN [*F*(1,19) = 12.221, *p* = *0.0*02] and RMSSD levels [*F*(1,19) = 7.302, *p* = *0.0*14], but there were no significant group effects for both HRV parameters.

### Correlations

General trait fatigue negatively correlated with SDNN (*r* =  − 0.375, *p* = *0.0*29) and RMSSD (*r* =  − 0.456, *p* = *0.0*07) during the second half of the vigilance task. Moreover, trait cognitive fatigue positively correlated with *median* reaction time during the first half of the vigilance task (*r* = 0.462, *p* = 0.008), and during the second half of the vigilance task (*r* = 0.532, *p* = 0.002). Similarly, state fatigue following the vigilance task positively correlated with median reaction time during the first half of the vigilance task (*r* = 0.415*, p* = 0.020), and during the second half of the vigilance task (*r* = 0.415, *p* = 0.020).

## Discussion

The aim of the study was to investigate whether one of two different biofeedback-supported relaxation exercises (DB versus PMR) can alleviate MS-related fatigue and whether changes of current (state) fatigue correlate with alterations in the autonomic nervous system. As a corollary, we also investigated if biofeedback interventions would elicit changes of ANS activity in MS patients. We found that (1) changes in biofeedback-supported autonomic nervous system activity induced by deep breathing can be objectively measured through HRV; (2) performing a vigilance task induces state fatigue; (3) increasing the feeling of fatigue through the vigilance task is associated with decreased parasympathetic components of HRV and recovering from fatigue is associated with a subsequent increase in these components; and (4) there is some evidence that PMR may be beneficial for fatigue recovery after the vigilance task, while DB is not.

There is evidence of autonomic dysfunction in MS patients with fatigue, more specifically, altered HRV [45]. Specifically, patients with high levels of fatigue have been found to exhibit greater autonomic dysfunction, including a reduced HRV that reflects parasympathetic activity. These has been shown by Sander et al. [16], whereby the group with high fatigue showed significantly lower SDNN and pNN50 scores than the group with weak to moderate fatigue. Moreover, Flachenecker et al. (2003) [46] found that a pattern of sympathetic dysfunction was more pronounced in patients with MS-related fatigue than in those with MS without fatigue. The grouping of patients with relapsing–remitting and progressive MS subtypes, the majority being relapsing–remitting, may have limited our results, as the burden of autonomic dysfunction has been shown to be higher in progressive MS patients [29–31]. Another limitation is the lack of knowledge on whether subjects had plaques in brain regions known to influence heart rate variability. Nonetheless, autonomic dysfunction was not the focus of our study—but rather the possibility to apply biofeedback to alleviate fatigue.

Our first goal was to show that in the case where an autonomic dysfunction may be present in MS patients, biofeedback could still be applied to improve teaching relaxation strategies and may help balancing autonomic activity in patients with an autonomic dysfunction. We found that MS patients show the expected autonomic response during the cardiovagal reflex test (i.e., after carrying out deep breathing exercises) (Fig. 3). That is, SDNN levels significantly increased while stimulating the cardiovagal reflex. This is consistent with a previous study showing that deep breathing results in increased short-term levels of SDNN along with intensified feelings of relaxation compared with spontaneous breathing [47]. The cardiovagal tests lasted a minute and not the conventional recording of 5 min because the practice of deep breathing may be rather challenging for a longer amount of time, and shorter recording periods of 10 s, 30 s, and 60 s for RMSSD [48–50] and SDNN [48] have been considered adequate**.** The absence of an effect on RMSSD during the cardiovagal reflex test may be explained by the shorter duration for recording than the conventional minimum period of 5 min [40]. We conclude that biofeedback exercises may be advised for MS patients as a means of relaxation.

Studies showing behavioral indicators of feelings of fatigue are few [41, 51]. Hanken et al. (2015) [41] emphasized in their review that fatigue can only be measured behaviorally by applying sustained cognitive tasks assessing alerting or vigilance. Vigilance tasks draw attention away from the environment, eliciting mind wandering. According to the model of Hanken et al. [41, 51], fatigue starts to interfere with processes that should be directed toward the environment especially during such monotonous tasks.

**Fig. 5:**
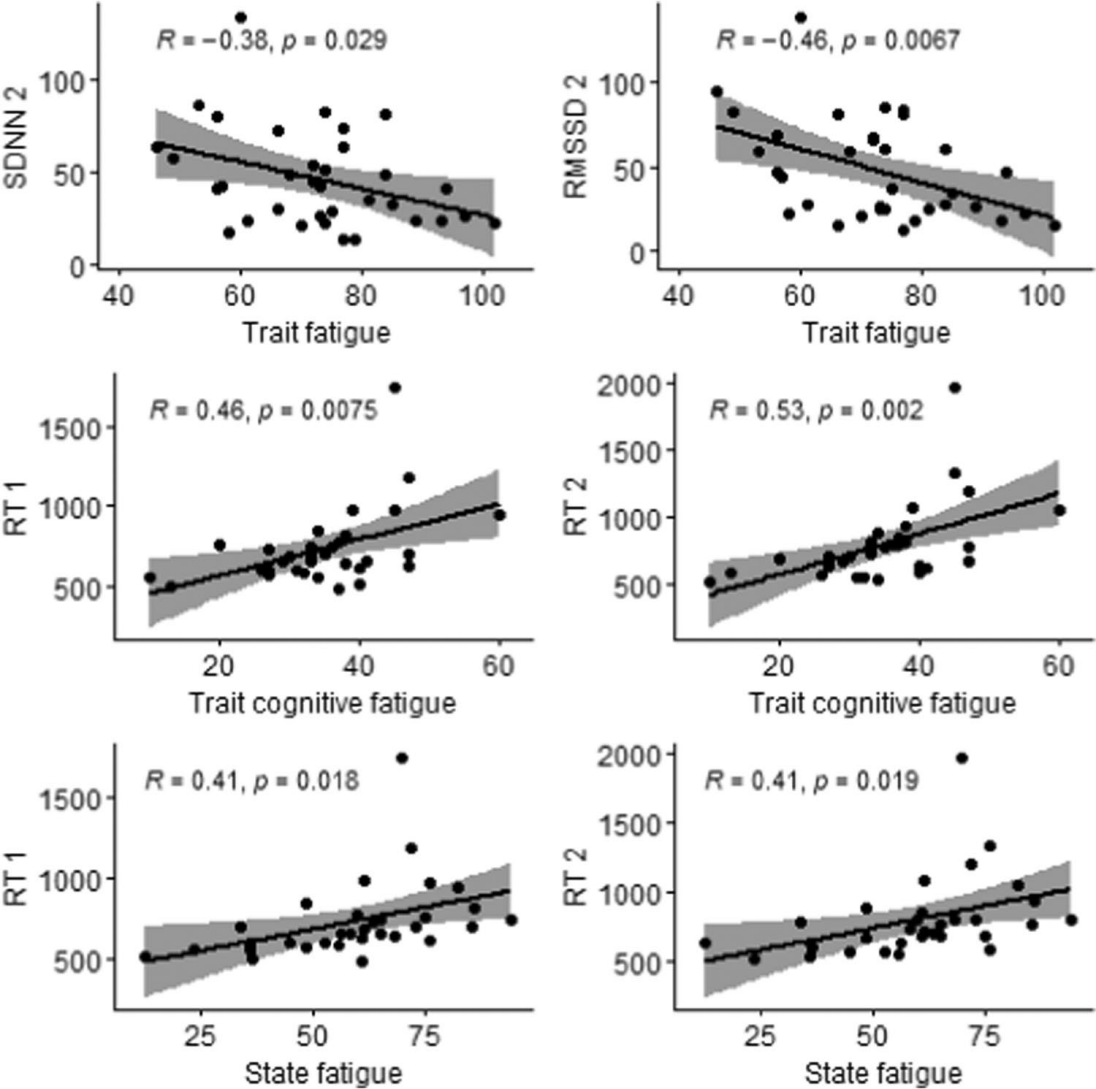
Correlation plots. *Note. RT* reaction time*; 1* first half of the vigilance task; *2* s half of the vigilance task

In this respect, our results demonstrate that the vigilance task can be reliably used to induce fatigue (Table 1, Fig. 4). Performing the vigilance task led to a highly significant increase in fatigue. This is in line with the study of Sander et al. (2020) [16] in which the vigilance task also led to increased feelings of fatigue. The notion that the vigilance task is suitable for inducing fatigue (by testing the immediate effects of relaxation groups on levels of fatigue) is endorsed by yet other findings from our study: Both trait and state fatigue correlated with a decreased *performance* (i.e., a longer reaction time) during the vigilance task (Fig. 5).

An attempt was made to measure autonomic nervous system activity in relation to mental strain during the vigilance task, by investigating whether HRV indices would change when participants are engaged in the task. We found that SDNN during the second half of the vigilance task was negatively associated with trait fatigue. We also found evidence that changes in fatigue correlate with alterations in the autonomic nervous system. SDNN increased significantly after recovery from the vigilance task and there was a marginally significant increase of RMSSD (Table 2). We have recently argued that HRV might be related to the feeling of fatigue in MS patients, due to vagal nerve signaling that controls heart rate and processes bodily inflammation to the brainstem and the hypothalamus, thus eliciting a mild form of “sickness behavior,” which encompasses fatigue as one of its core symptoms [45]. In this study we show that recovery from fatigue is associated with changes in HRV and/or vice versa, especially for relapsing–remitting patients, as the progressive MS groups were too small for a group specific analysis. Granting that this observation does not allow any causal interpretation, it still allows to speculate about the possibility of a relationship between vagal activity and subjective experience of fatigue.

The primary goal of our study was to compare two different biofeedback groups and their effect on fatigue in MS patients. Apparently, only PMR significantly improved patients’ fatigue level, while DB did not. We can only speculate as to why fatigue effectively decreased after PMR, and not after DB. Following the model of Hanken et al. (2014, 2015) [41, 51], fatigue draws attention to internal states and interferes with externally directed attention as soon as the focus of attention is allowed to wander. When the focus of an individual’s attention is predominantly allocated to fatigue, it becomes difficult to re-orient the attention again to external events. Now, a common characteristic of both relaxation techniques is that fatigue induced by the vigilance task could be alleviated by introducing a new focus of attention on bodily processes. However, both relaxation strategies are supposed to direct inward attention to the body. During the PMR exercise, distal body parts were likely the focus of attention, whereas DB may have directed the focus to the homeostatic state and autonomic functioning. In this behalf, PMR might have a higher potential to detract attention away from the feeling of fatigue.

Other investigators have also reported a positive effect of PMR in reducing fatigue for in MS [52] and in other chronic diseases such as chronic obstructive pulmonary disease [53, 54]. However, our goal was not to document a global effect of biofeedback on fatigue (as in almost all studies conducted on this topic), but to propose a short-term strategy for decreasing fatigue by refocusing attentional control, that could be implemented at any point during the day. Moreover, it should be taken into consideration that a direct comparison of state fatigue did not reveal any significant results in the PMR and DB groups, but only in intra-group comparisons of state fatigue. Therefore, attempts to explain possible differences between DB and PMR for controlling fatigue appear to be premature given the current empirical evidence.

The average level of fatigue of our total group was severe with an average FSMC total score of 72.7 ± 13.7, and only 9 patients scored below the cut-off score for severe trait fatigue. In the study by Sander et al. (2020) [16], PMR therapy enhanced parasympathetic nervous system activity, and these mainly concerned patients with weak to moderate levels of fatigue and not those with severe fatigue. A review on the efficacy of relaxation interventions indicated that these practices are not effective in patients with severe forms of several psychological disorders such as depression/dysthymia, anxiety disorders, alcoholism, and sleep disorders [55]. Including patients that suffer from lower levels of experienced fatigue might provide stronger results.

We measured state fatigue severity with a visual analog scale and did not track how long it took for fatigue to completely return to baseline levels. Visual analog scales are more sensitive to changes in fatigue states than fatigue questionnaires [56]. However, at the same time, they focus on short-term changes, which include spontaneous and reactive fluctuations like increasing tiredness, whereas fatigue questionnaires are designed to measure an enduring mental state, which per definition should be roughly related to environmental strain. Therefore, our results need to be replicated in a treatment study investigating the effect of learned strategies of short-term relaxation on fatigue that is being measured with questionnaires.

Drawbacks of our study are the small sample size, the lack of a healthy control group, and that only one session for instruction of the relaxation procedure was implemented. Future studies with a similar approach should include longer and more frequent PMR and DB exercises and focus on patients suffering from weak to moderate fatigue to uncover possible effects of DB on MS fatigue.

To sum up, we illustrate that engaging in PMR exercises may help alleviate symptoms of fatigue in persons with MS. If this holds true, short breaks of PMR after situations that evoke high fatigue during the day may help patients to recover faster and to decouple from the expectation of fatigue and the self-restriction in executed activities leading to a vicious circle of further deconditioning. Integrating short breaks of PMR may potentially improve their quality of life. Within this framework, using a vigilance task in combination with a measurement of HRV may help evaluate relaxation procedures as a treatment of fatigue.

## Data Availability

The dataset is now openly available in Figshare with the identifier 10.6084/m9.figshare.24101784.
